# The Impact of the Brain-Derived Neurotrophic Factor Gene on Trauma and Spatial Processing

**DOI:** 10.3390/jcm6120108

**Published:** 2017-11-27

**Authors:** Jessica K. Miller, Siné McDougall, Sarah Thomas, Jan Wiener

**Affiliations:** 1 Faculty of Human, Social & Political Science, University of Cambridge, Cambridge CB2 1TN, UK; 2 Department of Psychology, Bournemouth University, Poole BH12 5BB, UK; smcdougall@bournemouth.ac.uk; 3 Faculty of Health & Social Sciences, Clinical Research Unit, Bournemouth University, Poole BH12 5BB, UK; saraht@bournemouth.ac.uk; 4 Department of Psychology, Ageing and Dementia Research Centre, Bournemouth University, Poole BH12 5BB, UK; jwiener@bournemouth.ac.uk

**Keywords:** BDNF, Brain-Derived Neurotrophic Factor, navigation, spatial processing, trauma, trauma processing, Post-Traumatic Stress Disorder, PSTD, allocentric, hippocampus

## Abstract

The influence of genes and the environment on the development of Post-Traumatic Stress Disorder (PTSD) continues to motivate neuropsychological research, with one consistent focus being the Brain-Derived Neurotrophic Factor (BDNF) gene, given its impact on the integrity of the hippocampal memory system. Research into human navigation also considers the BDNF gene in relation to hippocampal dependent spatial processing. This speculative paper brings together trauma and spatial processing for the first time and presents exploratory research into their interactions with BDNF. We propose that quantifying the impact of BDNF on trauma and spatial processing is critical and may well explain individual differences in clinical trauma treatment outcomes and in navigation performance. Research has already shown that the BDNF gene influences PTSD severity and prevalence as well as navigation behaviour. However, more data are required to demonstrate the precise hippocampal dependent processing mechanisms behind these influences in different populations and environmental conditions. This paper provides insight from recent studies and calls for further research into the relationship between allocentric processing, trauma processing and BDNF. We argue that research into these neural mechanisms could transform PTSD clinical practice and professional support for individuals in trauma-exposing occupations such as emergency response, law enforcement and the military.

## 1. Introduction

Post-Traumatic Stress Disorder (PTSD) is an increasingly visible mental health issue that represents a considerable public health burden [1] across many civilian and professional populations. With mounting pressure on health, military and emergency response sectors (https://www.pdtrust.org/help/research/post-traumatic-stress/) to look after the psychological wellbeing of their staff in the face of unprecedented demand from major incidents and resource deficits, understanding PTSD has perhaps never been so critical. Fortuitously, neuropsychological research over recent years has also moved at a commensurate pace and in this paper, we seize the opportunity to reflect on the progress (and pitfalls) of that research. We review recent literature, present findings from exploratory research (provided in more detail in the Appendix A) and highlight design issues which may be key to understanding how genetic and environmental conditions interact to influence PTSD vulnerability, etiology and recovery. To do this, we look at another area of cognitive function—navigation—which may provide us with vital information about the resilience of a specific part of our brain (the hippocampus) on which we rely to process trauma exposure [2,3,4,5,6].

## 2. The Neural Basis of Post-Traumatic Stress Disorder (PTSD)

Contemporary theories of PTSD which have been developed from cognitive theories and clinical research [2,3,4,5,6,7,8,9] describe PTSD in the context of information processing. A predominant theory is that of dual representation [2,4,6,7,8]. Dual Representation Theory describes how trauma processing operates with two types of memory representations in the limbic system: those which are associative and those which are contextual [8]. Associative representations of trauma are typically involuntary, fear-based, and originate in the amygdala. Contextual representations, in contrast, are retrieved voluntarily and mediated by the hippocampus [10,11]. According to Dual Representation Theory, effective trauma processing involves applying context to the sensory and evocative experiences of trauma to consolidate them into long term memory and file them as “past”. Trauma literature often refers to egocentric, associatively conditioned responses to stimuli as being typical in cases of post-traumatic stress, and these responses can be described by a signature symptom of PTSD, the “flashback” [9,10,11,12,13]. Hippocampal representations, on the other hand, provide episodic and spatial context for extreme experiences, which enables individuals to make sense of when and where traumatic incidents occurred [14,15,16,17,18]. However, when the hippocampus is down-regulated (e.g., by trauma or stress) it is less able to contextualise or anchor traumatic experiences in space and time, allowing them to intrude in the present, thus prolonging the stress response [4,19,20,21,22,23,24].

## 3. Brain-Derived Neurotrophic Factor (BDNF)

Stressful or traumatic incidents in the environment are not the only causes of down-regulation in the hippocampus; genetics also has a substantial impact [1,5,24,25,26,27,28,29,30]. Identifying genes which are relevant to the development of PTSD has been a relentless motivator for numerous genome-wide association studies, twin studies and candidate gene studies [1,5]. A recent review [31] identified 25 such studies, many of which highlighted the specific role of a gene called Brain-Derived Neurotrophic Factor (BDNF). BDNF is expressed in the limbic system, moderating fear responses and broadly regulating the stress response [5,20,22,23,30,31,32]. It is also expressed outside the limbic system, such as in the retina, kidneys and prostate [33], and has been considered integral to critical periods of human development [34]. The BDNF gene codes for the BDNF protein which is then expressed to promote the growth and survival of neurons, particularly those in the hippocampus [26,31,35]. BDNF-related neuroplasticity is considered an important component in maintaining the integrity of the hippocampus [5,25,34,35].

However, this operation is complicated by the fact that the BDNF gene has two variants, derived from carrying “met” and “val” alleles, which differ in their functionality [5,15,18,20,24,25,29,31,32,35]. At a genetic level, allelic variation occurs at codon 66 on chromosome 11, resulting in an amino acid switch from valine (val) to methionine (met) and producing a val66met polymorphism which is unique to humans [18,31,35,36]. In the Caucasian population, 30% carry the met allele, either as the metmet homozygotes or valmet heterozygotes [35]. Typically, met carriers show less activity-dependent release of the BDNF protein in the hippocampus than val homozygotes [5,24,25,29,35]. This means that in met carriers (rather than val homozygotes) sufficient BDNF protein may not be released into the hippocampus for it to respond appropriately to the demands that the environment may place on it, such as the demand for consolidating traumatic experiences into long term memory [20,22,26,30,31,32,37,38].

Given the compounding effect of the BDNF polymorphism on hippocampal function, we would anticipate that post-traumatic stress would be more prevalent and severe in met carriers, if other environmental conditions have been controlled for. This appears to be borne out by Zhang et al.’s (2014) finding that PTSD was more prevalent and severe in met allele carriers [32]. Specifically, the study revealed that the allelic frequency of BDNF met was twofold higher in those with probable PTSD. In support of this finding, it has recently been proposed that sufficient BDNF release may be involved in helping to prevent PTSD because its operation induces fear extinction and ensures successful trauma processing [5,20,22,30,31,32,37,38].

It is worth noting from BDNF and PTSD studies [32] the importance of controlling for environmental conditions. Indeed, a failure to consider the demand on the hippocampus that different environmental conditions can present may account for mixed findings to date in studies relating BDNF to PTSD [1,31,32,36]. Nonetheless, in 2014, Zhang and colleagues [32] successfully controlled for these conditions and reported a direct relationship between the BNDF gene and PTSD in a population of U.S. military Special Operations personnel.

## 4. Hippocampal Function, Navigation and BDNF

**Figure 1:**
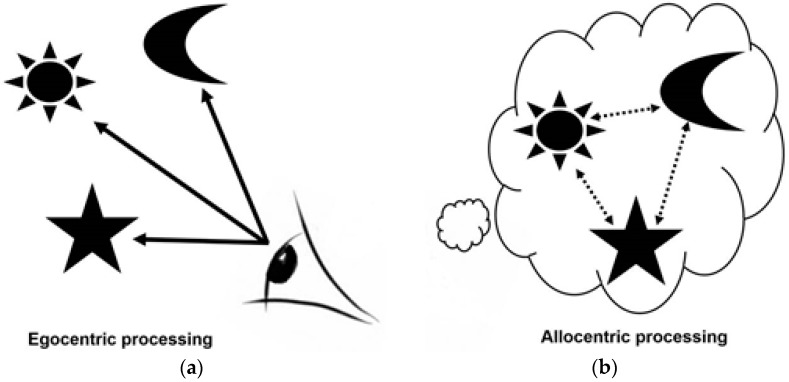
(**a**) Egocentric processing and (**b**) allocentric processing of spatial relationships.

Next, we consider how navigation can be used to assay hippocampal function [10,11,39,40,41]. Our situational awareness and our ability to orient ourselves and navigate our way through the world rely on two forms of mental representations, those that are hippocampal independent (egocentric representations) and those that are hippocampal dependent (allocentric, see Figure 1).

(i) Egocentric processing is viewpoint dependent and associative, relying on local landmarks in line of sight. This form of processing is not dependent on hippocampal processing: egocentric spatial memory representations are independent of the hippocampus, relying on the striatal circuit, whereas allocentric representations are thought to rely heavily on the hippocampal circuit [2,6,8,10,11,39].
(ii) Allocentric processing enables individuals to construct a viewer-independent representation of the relationship between objects /landmarks/ places in an environment. A spatial “map” [11] is created in which key landmarks are represented in relation to one another rather than in relation to the viewer. This form of representation is particularly important in route planning and is vital for contextualising information during navigation [10,11,28,29]. Allocentric processing, in contrast to egocentric processing, relies heavily on the hippocampal circuit [10,11,39].

So, given that the hippocampus facilitates (allocentric) spatial processing, individuals’ navigation skills depend on effective hippocampal function and can therefore act as an index of hippocampal integrity [5,6,8,10,23,41]. There have been very few studies examining the effects of BDNF on navigation. However, there is evidence in recent neuropsychological literature for a relationship between the BDNF gene and hippocampal dependent (allocentric) spatial processing. A study from 2011 by Banner et al. [29] provides supportive evidence that met-carrying BDNF genotypes rely more on hippocampal independent (egocentric) spatial processing to complete a navigation task than valval homozygotes (see also Lövdén et al., 2011, for a similar proposal based on a study with a much smaller sample) [28]. To demonstrate this, Banner et al. assessed participants’ spontaneous strategy use in a virtual maze. A higher proportion of BDNF metmet homozygotes spontaneously used egocentric strategies in comparison to valval homozygotes, whereas a higher proportion of valval homozygotes spontaneously used allocentric strategies. Both studies [28,29] made an explicit connection between less BDNF release in met carriers, lack of hippocampal engagement in spatial tasks and a bias toward implicit, associative spatial processing. In short, BDNF met carriers are generally considered to be more likely to engage in egocentric processing (which does not rely on the hippocampus) in comparison to valval homozygotes (see also [5,15,28,29,40]), who have greater access to effective allocentric processing via the hippocampus.

## 5. Bringing Together Allocentric Spatial Processing, the BDNF Gene and PTSD

**Figure 2:**
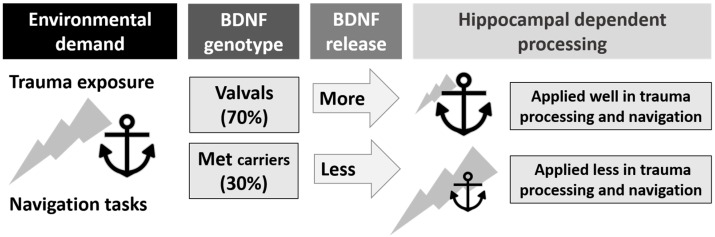
The relationship between Brain-Derived Neurotrophic Factor (BDNF) genotype, Post-Traumatic Stress Disorder (PTSD), hippocampal processing, and navigation skills (represented by the anchor). BDNF genotype influences activity-dependent release of the BDNF protein used in hippocampal processing of traumatic and spatial information, potentially placing some genotypes at a disadvantage for trauma resilience and navigation competence.

This focused literature review shows that the relationship between BDNF and hippocampal dependent processing and trauma is complicated, as illustrated in Figure 2.

With regard to trauma processing, severity and prevalence of PTSD is positively related to carrying the BDNF met allele [5,20,30,31,32,36,37,38]. With regard to spatial processing, there is evidence of an egocentric bias in BDNF met carriers but no clear differences in allocentric performance between BDNF genotypes were reported in either study [28,29]. Interestingly, egocentric bias in navigation strategy use and allocentric performance deficits have also been recently demonstrated in cases of PTSD (and trauma exposure) [2,3,6,8,40]. Neuropsychology has yet to bring these findings about BDNF, hippocampal dependent processing and trauma (or PTSD) together into one human experiment. A rodent model in 2007 [41] went so far as to demonstrate impaired spatial learning in the Morris Water Maze and significantly reduced extinction of conditioned fear in BDNF “knockout” rats. Although based on deleting rodent genes rather than genotyping human populations, this cross-discipline study stresses the possibility that cognitive spatial processing deficits and impairment in managing trauma exposure may be directly related to BDNF gene expression in the hippocampus.

In 2012, we sought to investigate how spatial processing impairment and trauma exposure processing may be related to BDNF genotypes in an exploratory extension of a human study (*n* = 150) which assessed the impact of PTSD on navigation [6,40]. *Full details of and data from the exploratory study are provided in the Appendix A*. Our intention was to determine if any bias in BDNF met carriers toward hippocampal independent spatial processing:

(a) was evident in the virtual environment navigation task being used in the main study [6,28,29,40],
(b) remained, when controlling for hippocampal down-regulation from PTSD [2,3,4,6,8,40,41,42], and/or
(c) correlated with subjective measures of self-reported navigation competence [43,44,45].

**Figure A1:**
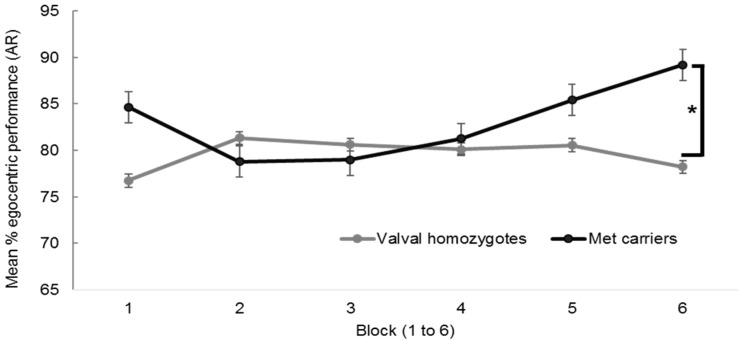
Mean egocentric performance on the Alternative Route (AR) paradigm between BDNF genotypes (*n* = 140, with *n* = 96 valval homozygotes and *n* = 44 met carriers) with standard error bars, showing significant performance differences in block 6, * *p* < 0.05.

In summary, in the diverse sample (*n* = 150) of civilian, police and military populations, PTSD severity and prevalence were similar across BDNF groups. In the sample population, 57 participants had probable levels of PTSD and of those without probable PTSD, 60 were trauma exposed and 33 were not. Participants’ navigation performance was assessed using the Alternative Route paradigm [6,10,46]. When graphed (see Figure A1 in the Appendix A), the data showed a distinctly divergent pattern of egocentric performance between BDNF valval homozygotes and met carriers, resulting in significantly higher egocentric performance in met carriers at the end of the navigation task, echoing interpretations of egocentric bias in met carriers in the previous studies [28,29]. Using self-report navigation questionnaires (specific questions from which had been shown to “predict” allocentric spatial processing in earlier studies [43,44,45]), we were also able to show for the first time that only BDNF valval homozygotes (not met carriers) were accurate in judging their own competence at allocentric spatial processing (see Table A1 in the Appendix A). Overall, our exploratory data were indicative of BDNF-related differences in hippocampal dependent and independent navigation behaviour, irrespective of PTSD. While interesting, it is important to note that these findings were limited by several design features. These limitations provide valuable insights for further research, and it is to those insights that we now turn.

## 6. The Future of BDNF Research

For candidate gene (BDNF) research to shape the future of clinical trauma interventions or to influence professional practices in occupations requiring situational awareness, studies need to be able to deliver accurate and ecologically relevant data [1,3,47,48]. Our exploratory research [40] into the relationship between BDNF, PTSD and spatial processing was limited by several factors. If these factors could be addressed in replication studies, significant progress in our understanding of *gene* × *environment* interactions in trauma and navigation could be imminent. Here, we briefly critique the design limitations of recent studies (including our own) and offer suggestions for improving data quality in key areas: experimental groups, performance measurement, subjective measures of navigation, and collection of further neurological data.

Sample populations for BDNF studies can be a contentious issue, with some traditional academic disciplines [30] typically favouring large cohorts (of thousands) and genome-wide association studies over the much smaller designs and sample sizes seen in candidate gene studies [1,36,42,47,48]. While some candidate gene study sample sizes have simply been too small (*n* < 20) to adequately represent the three BDNF genotypes (see [1,28,46]), other moderate samples (*n >* 100) (see [24,26,29,32]) have been able to demonstrate the influence of the gene on PTSD when other environmental conditions within and between experimental groups have been adequately controlled. This was a lesson learnt by Zhang et al. in 2014 [32], in their replication of an earlier study from 2006 [42] which did not control for trauma exposure type or severity, time since exposure or treatment status. Another important factor to control for in studies of hippocampal function is age [2,5,6,10,25,46]. A primary recommendation is for future BDNF and PTSD research to control for: age, time since exposure, treatment status and trauma exposure type or severity (at least distinguishing between civilian exposure and occupational exposure, such as the military or blue light services).

Navigation performance as a measure or index of hippocampal integrity is also key to BDNF research design. Identifying navigation tasks which produce data that can discriminate between hippocampal dependent and independent performance is challenging [6,8,10,11,40,41,46], yet vital. In our own study, the purity of the egocentric performance measure [6,40,46] was somewhat compromised by the fact that egocentric trials in the route learning task could feasibly be solved allocentrically. Some theorists may challenge this concern on the basis that egocentric processing is generally considered more parsimonious and therefore more likely a universal default means of solving simple tasks [49,50]. Nonetheless, implementing spatial processing measures which can accurately distinguish between allocentric and egocentric processing should remain a priority for any future studies which intend to compare functionality of the two memory systems. Similarly, disparity between studies which assess performance in allocentric and egocentric strategy use as opposed to allocentric or egocentric spontaneous strategy choice, is also something to be mindful of when comparing participants’ navigation behaviours [6,28,46,51]. Spontaneous navigation behaviour and navigation behaviour over the course of a learning paradigm likely measure different components of spatial processing and need to be clarified as such in research design. Asking participants directly how they think their behaviour may have changed over the course of a navigation task is a common approach and is it our recommendation that developing post-test self-report (i.e., think aloud) [28,29,43,46] measures may provide useful insights into how individuals think they navigate, even if this contradicts with their performance data.

Understanding individuals’ self-awareness of their ability to apply hippocampal dependent processing when required is not only valuable to research into the declarative hippocampal dependent memory system [26,28,29] but could be highly valuable for clinical trauma processing and professional navigation training interventions that rely on that form of information processing [2,3,6,7,9,13,23,39,40]. Developing more ecologically relevant subjective measures of spatial processing would benefit future BDNF studies greatly. The navigation questionnaire literature shows that the Santa Barbara Sense of Direction questionnaire [43] and selected questions from the Questionnaire of Spatial Representation [44] and the Fragebogen Räumliche Strategien [45] can predict navigation performance, yet the validity of the questions could be enhanced by introducing terminology and frames of reference more aligned with the types of spatial processing that sample populations may be familiar with on a day-to-day basis. For example, a subjective navigation measure for a military population could refer to topographical (landscape) changes from conflict as a frame of reference for certain questions. Whereas for policing populations, references to using satellite navigation while driving a response vehicle may be a more meaningful context. Using terms participants can relate to, they may increase task engagement, and their more focused self-reflection could enhance the validity of the self-report measures.

Finally, we consider the use of supplementary neurological data to support future research into the influence of BDNF on hippocampal processing. While research to date has clearly suggested a relationship between BDNF, hippocampal dependent processing, PTSD and navigation, the precise neural mechanisms underlying this relationship are as yet undefined. BDNF research has looked at volumetric measurement in the hippocampus using magnetic resonance imaging (MRI) and fluorescent microscopy [51,52,53], but whether volume differences are the result of BDNF-related protein release, neurogenesis, neuronal survival or synaptic plasticity is not clear [50,51,52,53,54]. Investigating these neural mechanisms is further complicated by the possibility that BDNF is released in response to different environmental conditions over the life span, meaning that age (or critical periods of development) and time since trauma exposure may need to be controlled for when investigating levels of BDNF in plasma, blood or saliva (as opposed to BDNF genotypes) [5,25,33,34,36,54,55,56,57,58].

Implementing a solid framework for future research into the relationship between hippocampal integrity, PSTD and spatial processing is likely a daunting, but we argue, necessary task. Such a framework could comprise combining:

(a) ecologically valid behavioural and subjective tests of navigation, supported by
(b) functional MRI (fMRI) and MRI-assessing activity or volume, possibly pre- and post-trauma exposure, with
(c) adequate neurochemical assessment of activity-dependent hippocampal BDNF release (for example, using blood serum) between genotypes, and
(d) closely matching participants to control for numerous differentiating variables, which may influence hippocampal and broader neuro degradation syndromes, ranging from age and environmental conditions of trauma exposure to epigenetic history and even nutrition [59].

## 7. Conclusions

Understanding the *gene* × *environment* interaction in relation to both trauma exposure and spatial processing has far-reaching practical, clinical and academic implications. In practical terms, if research could accurately quantify the contribution that carrying the BDNF met allele makes to an individual being able to successfully adopt hippocampal dependent information processing techniques, this could transform how trauma management and navigation training interventions are delivered in clinical and occupational settings. If 30% of the Caucasian (and up to 50% of non-Caucasian) populations [31,35,36,60] (i.e., BDNF met carriers) could access interventions that either deliberately encouraged hippocampal dependent processing or provided workable alternatives to hippocampal dependent processing, this could equate to a substantial improvement in those intervention outcomes. The contribution that research on the hippocampus has made to 21st century neuropsychology (epitomized by the awarding of the Nobel Prize for Physiology or Medicine to Professor John O’Keefe in 2014 https://www.nobelprize.org/nobel_prizes/medicine/laureates/2014/okeefe-facts.html) is well recognised. Further research into the BDNF gene would reinforce the value of understanding how the human hippocampus shapes our emotional and professional lives. Perhaps above all, we believe that developing this research will enable science and society to take an important step toward protecting the wellbeing and mental integrity of the hundreds of thousands of men and women who put themselves in the face of trauma as part of their everyday public service, in defence, emergency response and law enforcement. To do this, we need to embrace lessons from earlier (sometimes exploratory) research across the disciplines of genetics, trauma and navigation to ensure that as we move forward, we can offer neuroscience the caliber of data it needs to meet some pressing public issues head on.

## Figures and Tables

**Figure A2:**
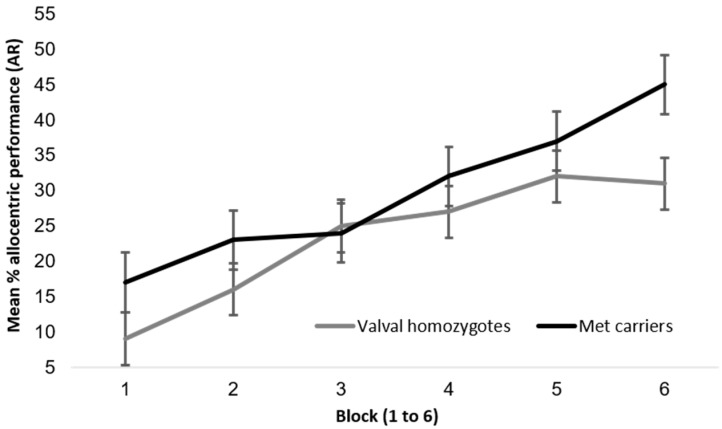
Mean allocentric performance on the AR paradigm between BDNF genotypes (*n* = 140, with *n* = 96 valval homozygotes and *n* = 44 met carriers) with standard error bars.

## Appendix A. Exploratory Research Data

The appendix contains details and data from the exploratory research into the Brain-Derived Neurotrophic Factor (BDNF) gene, undertaken as part of a study into the impact of trauma and Post-Traumatic Stress Disorder (PTSD) on navigation behaviour [5,6,40,46].

### Appendix A.1. Participants

The study sample population (*n* = 150) was diverse and was recruited from a university volunteer scheme (*n* = 83), two psychotherapy treatment centres (Traumatic Stress Clinic, Camden and Islington NHS) and the Intensive Psychotherapy Treatment Centre (Dorset Healthcare University NHS Foundation Trust) (*n* = 10), two police forces (*n* = 26) (Dorset and Cambridgeshire constabularies), and a military veteran treatment centre for PTSD (Combat Stress, Tyrwhitt House, Leatherhead, Surrey: registered charity #2060002) (*n* = 25 plus *n* = 6 staff). PTSD symptom severity and prevalence rates were measured using the PTSD Diagnostic Scale (PDS) [61]. Saliva samples for BDNF genotyping were collected using DNA *Genotek Orangene*™ self-test kits (DNA Genotek, Ottawa, ON, Canada). Participants were grouped according to whether they had been exposed to trauma or not, and if they had, whether or not they had clinical or probable levels of PTSD, using the PDS [46,61]. Sample sizes for PTSD were modest for the purpose of analysis by BDNF genotype. Of the 150 participants, 57 had probable levels of PTSD and of those who did not, 60 were trauma exposed and 33 were not trauma exposed. The frequency of the genotypes did not differ between experimental groups (*Trauma Unexposed, Trauma Exposed No PTSD*, and *PTSD*) for either the valval group, χ^2^ = 0.24, *p* = 0.88 or the “met carrying” group, χ^2^ = 0.04, *p* = 0.98. An independent samples *t*-test revealed no differences in PDS scores between BDNF groups, *t* (54) = −1.23, *p* = 0.23. This enabled the analysis of the influence of the BDNF gene to control for PTSD. The findings supported Zhang et al.’s conclusion in 2014 [32] that in order to demonstrate BDNF-related group differences in PTSD prevalence or severity, other environmental conditions may need to be controlled for (such as time since and severity of exposure, previous trauma and treatment access). It is important to note that this study could not assess the relationship between BDNF, spatial processing and PTSD, rather the relationship between BDNF and spatial processing.

### Appendix A.2. Methods

DNA saliva samples were genotyped for one single nucleotide polymorphisms (SNP): rs6265 (Val66Met), using a Taqman^®^ allele discrimination assay. The genotyping information acquired described which BDNF alleles were carried by the (anonymous) participant, i.e., whether they carried only the val allele (and were therefore valval homozygotes), both the val allele and the met allele (and were therefore valmet heterozygotes) or if they carried only the met allele (and were metmet homozygotes).

Allocentric and egocentric performance were assessed using a navigation paradigm, the Alternative Route (AR, [46]) which required participants to learn a route through a virtual environment. The task involved testing participants on their ability to re-join a route from the same direction and different directions from which they learned it. There were six blocks to the task. Mean performance on trials which involved joining the route from the same direction (same direction trials) provided a measure of egocentric performance. Mean performance on trials which involved joining the route from a different direction to the route as it was learned (different direction trials) provided a measure of allocentric performance. An ANOVA was used to assess BDNF group differences (met carriers vs. valval homozygotes) in egocentric and allocentric performance on a navigation task. Planned contrasts were then made at individual block level (1–11) using *t*-tests (see [6,40,46]).

Participants’ self-reported navigation competence was assessed using validated questionnaires; the *Santa Barbara Sense of Direction* (SBSOD) [43] and two items from the *Questionnaire of Spatial Representation* (QSR) [44] that target allocentric processing. Correlation analysis was used to analyse the relationship between self-reported competence and navigation performance within the BDNF groups (met carriers and valval homozygotes).

### Appendix A.3. Results

#### Appendix A.3.1. BDNF and Hippocampal Independent Performance

A repeated measures 2 × 6 ANOVA with the dependent variable egocentric performance, the between factor BDNF group (met carriers vs. valval homozygotes) and the within factor block (1 to 6) revealed no significant main effect of block, *F* (4.49, 138) = 0.90, *p* = 0.48, η*p*^2^ < 0.01, nor BDNF group, *F* (1, 138) = 0.99, *p* = 0.32, η*p*^2^ < 0.01, but a significant group × block interaction, *F* (4.49, 138) = 2.48, *p* = 0.03, η*p*^2^ = 0.02, visible in the divergent pattern in performance in Figure A1.

Planned contrasts using independent samples *t*-tests revealed met carriers performed better than valval homozygotes in the final block 6 (89% SD ± 17% vs. 78% SD ± 28%), *t* (138) = 5.65, *p* = 0.006 (equal variances not assumed). Applying Bonferroni’s correction (0.05/6 blocks) would require a *p* value of 0.008.

Despite the lack of significance in the overall BDNF group and egocentric performance interaction, closer examination of the data was undertaken to understand these final performance differences in block 6 and to look for any plausible explanation (other than chance) for the inverted curves in egocentric performance between the BDNF genotypes. The data showed there to be a statistically significant quadratic (rather than linear) effect of BDNF group, *F* (1, 138) = 5.59, *p* = 0.02 which supports earlier hypotheses for met carrier status explaining differences in egocentric navigation strategy preference over the course of the task [28,29,62].

Allocentric performance was comparable between BDNF genotypes, as found by both Banner et al. and Lövdén et al. in 2011 [28,29] and as illustrated in Figure A2. A repeated measures 2 × 6 ANOVA with the dependent variable (DV) of allocentric performance, the between factor BDNF group and within factor block (1 to 6) revealed a significant main effect of block (with performance increasing by block), *F* (4.07, 138) = 27.2, *p* < 0.01, η*p*^2^ = 0.17, but no significant main effect of BDNF group, *F* (1, 138) = 2.20, *p* = 0.14, η*p*^2^ = 0.02, and no significant interaction *F* (4.07, 138) = 1.71, *p* = 0.14, η*p*^2^ = 0.01.

#### Appendix A.3.2. BDNF and Self-Reported Navigation Competence

The final part of the analysis was to assess the navigation questionnaire data in relation to BDNF genotype. Participants’ scores for general self-reported competence in navigation (SBSOD total score) and the allocentric targeted QSR questionnaire items were positively correlated with their performance in allocentric navigation in the AR task. As illustrated in Table A1, only valval homozygotes’ self-reported competence positively correlated with their performance in allocentric navigation, suggesting that met carriers were less able than valval homozygotes to accurately describe their capacity for hippocampal dependent (allocentric) spatial processing.

**Table A1:** Pearson’s correlations (r) between Questionnaire of Spatial Representation (QSR) allocentric items, the Santa Barbara Sense of Direction (SBSOD) and allocentric performance in the AR paradigm in BDNF valval homozygotes (*n* = 102) and met carriers (*n* = 45), *p* < 0.01 **, *p* < 0.05 *.

Self-Reported Navigation Competence	Allocentric Performance in BDNF Valval Homozygotes (*n* = 102)	Allocentric Performance in BDNF Met Carriers (*n* = 45)
General competence (SBSOD)	0.26 *	0.05
Allocentric competence (QSR)	0.28 **	0.03
